# Supplementary material for: Integrated Proteomics and Metabolomics Link Acne to the Action Mechanisms of Cryptotanshinone Intervention
Source: Front Pharmacol. 2021 Sep 1;12:700696. doi: 10.3389/fphar.2021.700696 (PMC8440807; doi:10.3389/fphar.2021.700696)
Supplement: Supplementary file 1 [file DataSheet1.ZIP › Supplementary table 1-10 and material 1-3/Material 3-Metlib-MSMS/NEG-Metlib-MSMS/Metlib-MSMS/M452T85_forward/0.979,1-Palmitoyl-2-hydroxy-sn-glycero-3-phosphoethanolamine,(M-H)-.pdf]

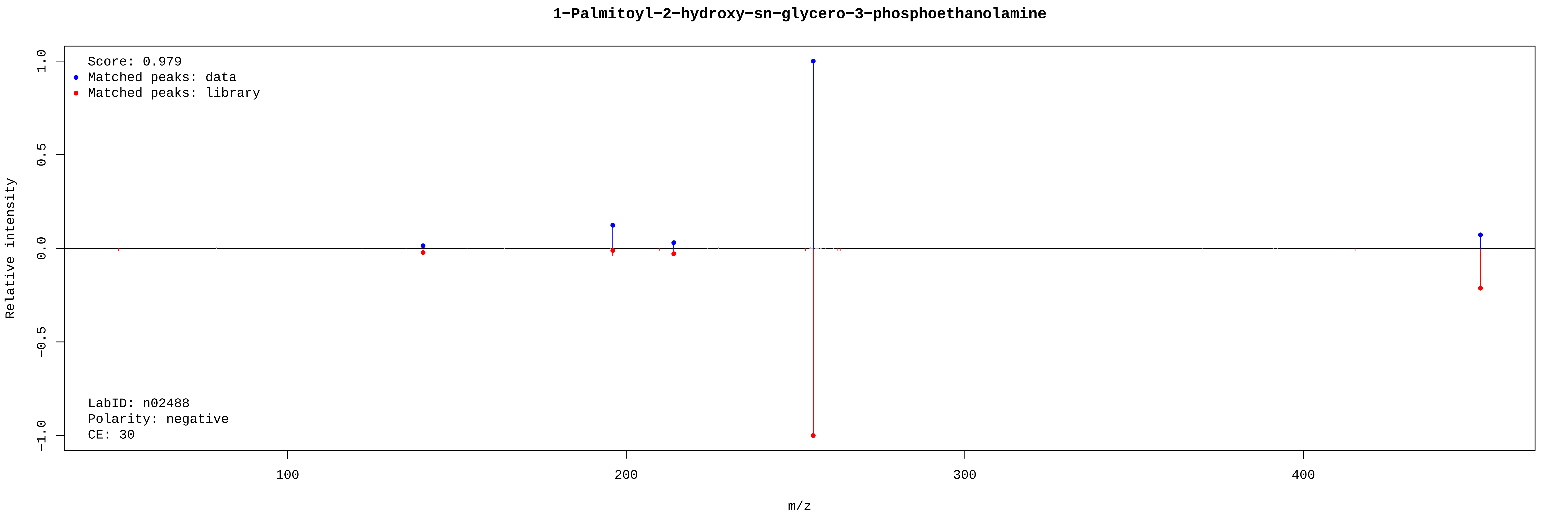

1-Palmitoyl-2-hydroxy-sn-glycero-3-phosphoethanolamine
Score: 0.979
Matched peaks: data
Matched peaks: library
LabID: n02488
Polarity: negative
CE: 30
Relative intensity
1.0
0.5
0.0
-0.5
-1.0
100
200
300
400
m/z
